# Thirty-Five-Year History of Desialylated Lipoproteins Discovered by Vladimir Tertov

**DOI:** 10.3390/biomedicines10051174

**Published:** 2022-05-19

**Authors:** Victor Glanz, Evgeny E. Bezsonov, Vladislav Soldatov, Alexander N. Orekhov

**Affiliations:** 1 Laboratory of Cellular and Molecular Pathology of Cardiovascular System, Federal State Budgetary Scientific Institution “Petrovsky National Research Centre of Surgery”, 117418 Moscow, Russia; vito-mail2012@yandex.ru; 2 Laboratory of Angiopathology, Institute of General Pathology and Pathophysiology, 125315 Moscow, Russia; 3 Department of Biology and General Genetics, I. M. Sechenov First Moscow State Medical University (Sechenov University), 105043 Moscow, Russia; 4 Pharmacology and Clinical Pharmacology Department, Belgorod State National Research University, 308015 Belgorod, Russia; soldatov_v@bsu.edu.ru

**Keywords:** atherosclerosis, low-density lipoproteins (LDL), sialidase, trans-sialidase, desialylation, atherogenicity

## Abstract

Atherosclerosis is one of the leading causes of death in developed and developing countries. The atherogenicity phenomenon cannot be separated from the role of modified low-density lipoproteins (LDL) in atherosclerosis development. Among the multiple modifications of LDL, desialylation deserves to be discussed separately, since its atherogenic effects and contribution to atherogenicity are often underestimated or, simply, forgotten. Vladimir Tertov is linked to the origin of the research related to desialylated lipoproteins, including the association of modified LDL with atherogenicity, autoimmune nature of atherosclerosis, and discovery of sialidase activity in blood plasma. The review will briefly discuss all the above-mentioned information, with a description of the current situation in the research.

## 1. Introduction: Atherogenicity Phenomenon and the Concept of Modified LDL

The diseases with atherosclerosis at their roots are responsible for the majority of death cases in developed and developing countries. Despite certain progress that has been reached in diagnostics, pharmacological intervention related to the lowering of blood cholesterol levels and surgery, there is still no “universal” method or therapy approach that would provide efficient and robust results in the prevention and curing of atherosclerosis. The reason for such a tragedy for millions of patients, from the author’s point of view, lies in the interdisciplinary character of this pathology (mostly it is not placed as a separate subject). Atherosclerosis is studied in numerous medical fields including cardiology, angiology, neurology, hematology, etc. As a result, patients receive mostly symptomatic treatment; the knowledge obtained in different fields is not efficiently distributed between these fields, since there is no unifying power that would combine all the results obtained together in order to find efficient and reliable approaches to prevent and reduce atherosclerotic lesions in arterial walls based on a deep understanding of the pathological changes that happen during atherosclerosis initiation and its further development. The separation of atherosclerosis from its clinical manifestations would allow one to focus the attention on the pathological process, and, as a result of the study of such a process, to develop new approaches for detection, prevention and curing of this disease.

Atherosclerotic pathological changes are related to the appearance of atherosclerotic lesions in arterial walls. Many diseases are connected to atherosclerosis due to a rapid decrease in blood flow caused by a decreased vessel lumen [1]. The arterial wall consists of the following three envelopes: 1. the closest one to the arterial lumen is called intima (this is where atherosclerotic lesions develop); 2. the medium envelope is called media; 3. the external envelope, the most distant from arterial lumen, is called adventitia.

Atherosclerotic plaque (a local thickening of intima protruding into the arterial lumen) serves as the main reason for disrupted circulation in vital organs in the case of atherosclerosis. In general, atherosclerotic plaque is characterized by elevated levels of oxidized lipoproteins, triglycerides, oxidized cholesterol products, and free fatty acids. The protein components are apolipoprotein A1 (apoA1), fibrinogen, arylesterase-1 (PON-1), and clusterin. If plaques become unstable in the carotid artery, this means a significantly higher probability of ischemic events. Plaque formation and its structural alterations are mediated by direct interaction between plaque components and various circulating elements in blood [2].

The multiple mechanisms of atherosclerotic plaque formation, including lipoprotein transfer and lipoprotein modification, can be summarized as atherogenicity, a known phenomenon since 1986. Atherogenicity is opposed by antiatherogenic agents, such as blood serum high-density-lipoprotein cholesterol (HDL-C), paraoxonase, and PON1 [3]. Another atherogenic factor is the plasma cholesteryl ester transfer protein (CETP), which affects the distribution of cholesteryl esters between HDL and low-density lipoproteins (LDL) [4]. In 1986, it was demonstrated for the first time that blood serum from coronary heart disease (CHD) patients possesses atherogenic potential. The serum from such patients facilitated lipid accumulation in cultured human aorta subendothelial cells, which showed no such effect when treated with serum from healthy individuals [5]. Inflammation also contributes to atherosclerosis-related pathological changes (in addition to lipids), which highlights the complexity of this disease [6].

LDLs play a major role in atherosclerosis development and in ischemia. These lipoproteins are ApoB-containing particles, alongside very low-density lipoproteins (VLDLs), intermediate density lipoproteins (IDLs) and chylomicrons [7]. LDLs carry out the transport of lipids mediated by LDL receptors [8]. Small and dense LDL are more prone to oxidation and possess a higher affinity for proteoglycans.

The main atherogenic modifications of LDL are oxidation [9], and enzymatic modifications. These changes result in the formation of pro-inflammatory LDL. The contribution of oxidation to the LDL modification spectrum, which was traditionally thought of as a primary mechanism, is now met with opposing experimental evidence [10]. For example, it was shown that oxidation alone is not sufficient to promote the uptake of LDL by macrophages, but that there are receptor-independent ways of LDL accumulation. This can be attributed to fluid-phase pinocytosis of LDLs mediated by protease calpain-6. [11]. Cytotoxic enzymatically modified LDLs, referred to as minimally modified (MM) LDLs, are supposedly first (along with native LDLs) to enter the arterial intima during atherogenesis, where LDL can be oxidized due to the inaccessibility of circulation [12].

The components of the pathway involved in the clearance of LDL from blood, such as LDL receptor (LDLR), and proprotein convertase subtilisin/kexin type 9 (PCSK9) responsible for the enhanced degradation of LDLR (also related to increased damage of mitochondrial DNA, and certain types of cancer), should, therefore, be considered as factors affecting atherogenicity [13,14,15].

The aim of this review is to summarize the current understanding of the role of desialylated proteins in atherosclerosis development and provide future directions of the research in this field.

## 2. Desialylated LDL as Atherogenicity Factor and Its Role in Atherosclerosis

It was quite logical to assume that modified LDL capable of inducing lipid accumulation in the cells of arterial walls should be looked for, first of all, in the blood of patients that already have their vessels subjected to atherosclerotic influence. In order to test this idea, a fraction of total LDL was purified from the blood plasma of healthy individuals and for the patients with atherosclerosis, angiography was used. The initial culture of subendothelial cells purified from human aortae was used to test the ability of purified LDL to induce intracellular accumulation of lipids (i.e., exactly the cells that accumulate lipids upon atherosclerosis), with the finding that LDLs from healthy individuals were not able to induce intracellular accumulation of phospholipids or neutral lipids in most of the cases studied, while LDLs from patients with coronary atherosclerosis were capable of inducing a 1.5-fold accumulation of free cholesterol and triglyceride content, and a 1.5–5-fold accumulation of esterified cholesterol in the cells cultivated [16,17].

The identification of the chemical composition of the LDLs isolated from healthy individuals and patients with atherosclerosis did not show any differences in the content of protein, phospholipids, triglycerides, free, and esterified cholesterol. Interestingly, the total content of sialic acid in LDL from the patients with carotid atherosclerosis was shown to be 2–3-fold lower than the one of healthy individuals [18,19].

It is known that sialic acid residue is a terminal monosaccharide in an asparagine-linked complex bi-antennae carbohydrate of ApoB protein of LDL. The next residue following sialic acid is galactose monosaccharide, which would become a terminal residue after the removal of the sialic acid residue. This information was used for the purification of desialylated LDLs using lectin specific for terminal galactose (agglutinin from *Ricinus communis*) in affinity chromatography. The desialylated LDL fractions purified using this approach were shown to contain 2–3-fold less of total sialic acid than the fraction of sialylated LDL, which was not retained during chromatography, confirming the validity of such an approach for the separation of sialylated and desialylated LDL [20].

The following development of this approach (column lectin-based chromatography), in combination with solid-phase lectin enzymatic assay, allowed the estimation of the content of desialylated LDLs in the blood of patients with atherosclerosis as belonging to the range from 20 to 60%, while the one for healthy individuals usually did not exceed 10% [21].

Incubation of the fraction of sialylated LDLs with subendothelial cells isolated from the intima of human aorta did not affect the intracellular content of phospholipids and neutral lipids [20,22], while desialylated LDLs eluted from the lectin column with 50 mM of galactose on average caused a 1.5–2-fold accumulation of unesterified cholesterol, and triglycerides, and 2–7-fold accumulation of cholesterol esters in the cells cultivated [20,22]. It can be concluded that the desialylated fraction of human LDL is atherogenic, and sialylated LDLs do not possess such a property, and should be considered as native non-modified LDL.

Further studies showed that glycoconjugates of the ApoB protein in human LDL consist of N-acetyl-glucosamine, galactose, mannose, and sialic acid in a molar proportion of 2:1:2.5:1 [23]. The content of N-acetyl-glucosamine, galactose, and mannose did not differ in protein-bound glycoconjugates from native sialylated LDL and circulating modified LDL of healthy individuals. The level of N-acetyl-glucosamine in ApoB protein from native LDL and circulating modified LDL from patients with coronary atherosclerosis did not differ from the corresponding fractions of LDL isolated from healthy individuals. The galactose and mannose content in circulating modified LDL from the patients was significantly lower than in native LDL [23].

The content of sialic acid in circulating modified LDL from healthy individuals was 15–30% lower than the one in native LDL. The content of sialic acid in the ApoB protein of native LDL isolated from the patients was approximately the same as the one from native LDL of healthy individuals. The content of sialic acid in protein-bound glycoconjugates of circulating modified LDL was 2–3-fold lower than the one for native LDL [23].

Thus, it was discovered that the atherogenic properties of blood serum are modulated by desialylation. Desialylated LDL, but not native LDL, promoted the accumulation of lipids in aortic intima cell culture [18]. Sialic acid belongs to negatively charged amino sugars and is an important component of native LDL. Sialic acids are terminal carbohydrate residues in apolipoprotein, and glycolipid components of HDL, LDL, VLDL and IDL. Sialic acids are predominantly associated with ApoB100, ApoA, ApoE, Apo (a), and ApoC. The role of sialic acid metabolism in disease is studied extensively, providing support of these findings.

The diminishing of arterial endothelium anion density due to desialylation leads to intimal thickening, as a certain level of sialylation has to be maintained, serving as a protective barrier [24]. Glycosylation is also required for the stable expression of LDL receptors [25].

The following two concepts of lipoprotein modification in the context of atherosclerosis exist: oxidation (which is more popular) and enzymatic modifications [26]. Enzymatic modifications of LDL may play a more important role in atherogenesis than oxidation. It is supported by the fact that enzymatically modified LDLs (E-LDL) tend to fuse and form larger particles, they convey complement-activating activity via a scavenger receptor-dependent pathway; they are more susceptible to be taken up by macrophages, leading to foam cell formation [27]. Desialylation can predispose LDLs to further modifications via a biochemical cascade that begins with cholesteryl esters hydrolysis, resulting in free cholesterol deposition [26]. Thus, atherogenicity of LDL desialylation is manifested through the changes in lipoprotein properties.

The sialic acid content of lipoproteins may affect the interaction of lipoproteins with proteoglycans; the removal of sialic acid residues from LDL via a neuraminidase treatment led to the increased formation of complexes between LDL and proteoglycans [28].

Desialylation reduces size and increases density of LDL particles [29]. Desialylated LDLs have higher triglyceride and fatty acid contents than native LDLs and have different structures of carbohydrate, lipid, and protein components [30]. Interestingly, the degree of ApoC-III sialylation in LDLs correlates with atherogenicity, as desialylated apoC-III0 isoforms are positively associated with proatherogenic lipid profiles [31]. Desialylated LDLs acquire negative charge [10,32]. This can be the result of not only desialylation, but of lipid loss, particle size reduction and peroxidation [33]. However, there is a report that sialic acid can still be found in electronegative LDL, and it was even predicted to contribute to the negative surface charge of these molecules [34]. More experiments should be conducted in the future in order to clarify these different observations. Negatively charged, or electronegative, LDLs that are able to trigger the aggregation of other LDL fractions are pro-atherogenic [35,36].

## 3. Autoimmune Nature of Atherosclerosis

Electronegative LDLs can initiate an immune response, resulting in anti-LDL autoantibodies and immune complex (IC) production [37]. Contrary to the originally postulated primary role of oxidized LDLs as a source of autoantibody both in atherosclerosis and in healthy individuals [38], it was demonstrated that in coronary atherosclerosis, autoantibody levels are significantly higher [39], and that anti-LDL antibodies bind preferably to desialylated LDL, which contradicts the originally proposed major role of oxidized LDLs in atherosclerosis autoimmunity. Moreover, immune complexes comprising of modified LDL and anti-LDL antibodies accelerated cholesterol, collagen and glycosaminoglycan accumulation in cell cultures of vascular endothelium, deposition of the extracellular matrix (ECM); stimulated the transformation of macrophages into foam cells. Foam cells are extremely immunogenic. Modified LDLs initiate an immune response by being taken up by antigen-presenting cells, which present LDL to T-cells, which leads to immune response amplification. Repeated exposure to the antigen makes T-cells produce cytokines and trigger inflammation [40].

LDL-immune complexes are taken up by macrophages via Fcγ-receptors. The size of the immune complexes ultimately determines their fate, as large ones are delivered to the spleen and liver, while soluble small-sized ICs remain in circulation. The pro-atherogenic properties of ICs can be countered either by pharmaceutical intervention [41] or by altering the glycosylation status of anti-LDL antibodies via targeting the Fc domain of the receptors. Sialylation of IgG decreases IC immunogenicity and reduces cardiovascular risk.

Circulating LDL-CICs are found in atherosclerotic lesions where they induce the accumulation of extracellular matrix in the vascular wall and cholesterol uptake as macrophages prime them to foam cells [42]. The unique properties of desialylated LDLs (increased atherogenic potential and immunogenicity) may be the promoting factor in anti-LDL antibody production. Anti-LDL antibodies have a low affinity to non-atherogenic native LDLs, generating LDL-CICs, which promote atherosclerosis [3]. The experimental evidence suggests that in cell cultures, LDL-CICs increase the cholesterol content within smooth muscle cells of the arterial intima. The selective removal of LDL-CICs from circulation makes non-bound circulating LDLs less atherogenic. A study of LDL-CIC properties showed that immune complex-bound LDLs have lower sialic acid content, as well as less phospholipids and neutral lipids. Immune complex-bound LDLs were more electronegative, smaller in size and had a higher density, thus demonstrating key properties of desialylated LDL.

Since atherosclerosis is an autoimmune disease that triggers an immune response to self-antigens, it is logical to assume that vaccines may potentially be beneficial in cardiovascular disease prevention, reducing inflammatory response by restoring tolerance to antigens. Most experimental studies in the field focused on developing a vaccine using lipoproteins and heat shock proteins (HSP) epitopes. Typically, T cells that react to LDL and HSP are eliminated in the thymus. LDL modification creates molecular mimicry via generating novel antigens, leading to persistent T cell reactivity and chronic inflammation [40].

It also should be noted that modified LDLs are capable to induce signals through toll-like receptors, which also play a role in innate immunity [43].

## 4. Multiple LDL Modifications

**Figure 1:**
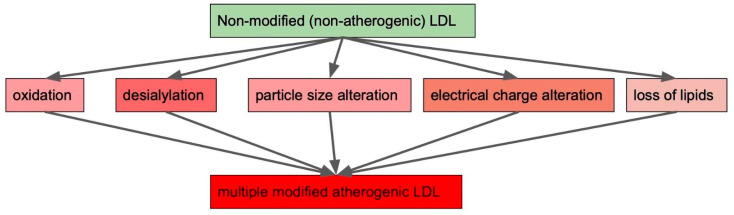
Possible modifications leading to the appearance of multiple modified low-density lipoproteins (LDLs) possessing atherogenic properties.

We already mentioned various ways through which LDL in circulation can be modified. These are oxidation, desialylation, particle size alteration, electrical charge alteration and loss of lipids (Figure 1). It needs to be noted that LDL modification types do not exist separately from each other, but form a cascade ultimately leading to atherogenesis. LDL modifications occur throughout the entire course of disease progression and give rise to different forms of modified LDL, contributing to cardiovascular metabolic and autoimmune diseases [12,44,45].

## 5. HDL Modifications

Optimal HDL and HDL-cholesterol (HDL-C) levels are required for active antiatherogenic processes and a low CVD risk. In healthy people, HDL accepts free cholesterol via ApoA1. ApoA1 modifications give rise to dysfunctional proatherogenic HDL [46]. A high CVD risk is associated with decreased HDL capacity to carry out the reverse transport of cholesterol, high levels of HDL oxidation, high content of triglycerides in HDL particle cores and smaller particle HDL size [47]. Dysfunctional HDL can be formed as a result of structural changes, post-translational modifications or lipid alteration. Oxidation and glycation are the most common post-translational modifications [48]. HDL oxidation occurs during inflammation with participation from myeloperoxidase MPO, which is shown to be excessively expressed in atherogenesis. HDL glycation occurs due to lipid and protein exposure to carbohydrates, as it happens in hyperglycemia. The glycome of HDL was extensively studied by Huang et al. [49]. It was shown that Hexose5HexNAc4Neu5Ac2, possessing two sialic acid residues, is the most abundant glycan in HDL. Interestingly, it is also widely present in blood. Thus, sialic acid contributes to HDL negative charge. Sialylated glycans in HDL proteins partake in interactions between molecules and cells, affecting HDL functionality. Sialylated ApoE is necessary for it to be recognized by HDL3 particles. In addition, the desialylation of Apo is associated with elevated levels of circulating *Streptococcus pneumoniae* neuraminidase, meaning that bacterial (and probably viral) infection may govern HDL desialylation in vivo. Fucosylated N-glycans levels were relatively low. Desialylation acts on HDL the same way as it does on LDL. Desialylated HDLs tend to lose their anti-atherogenic properties via impairing HDL capacity to remove cholesterol from arterial cells. Desialylated ApoE less actively integrates itself in HDL, inhibiting its anti-atherogenic properties [50,51]. In CVD, HDLs are distinctively modified. They contain less ApoA1 (but increased levels of oxidized ApoA1), ApoA2 and ApoE and are enriched in complement-activating C3 [52]. The desialylation of ApoE noticeably decreases its binding to HDL, thus impairing the antiatherogenic properties of HDL. ApoE terminal glycosylation is suggested to protect against spontaneous self-aggregation in atherosclerosis [53]. This was also observed in metabolic syndrome patients, where ApoC3, ApoE and SAA4 were less sialylated compared to the healthy control [54]. The association of lipoprotein desialylation with disease is not exclusive to LDL. A high content of desialylated HDL was observed in hemodialysis patients and in metabolic syndrome patients, linking the loss of sialylation to metabolic dysfunction [55]. Despite the established role of HDL and its modifications in health and disease, targeted interventions have yet to be proved beneficial [56].

## 6. Plasma Sialidase Activity

Sialidases are exoglycosidases in vertebrates. Sialic acid residues are often found at the terminal positions of the glycoconjugate chains of the cellular glycocalyx. Sialidases or neuraminidases catalyze the removal of these residues, thereby, modulating various normal and pathological cellular activities. The following four types of mammalian sialidases have been described: Neu1, Neu2, Neu3, and Neu4, which are encoded by different genes and are characterized by different subcellular localizations [57]. Atherogenic modified LDLs are recognized by scavenger receptors (SR), unlike native LDLs, which interact with LDL-R. SR and Siglec-1, a macrophage-specific pattern recognition receptor, are known to participate in viral and bacterial pathogen recognition, meaning that LDL recognition and viral infections act as stimuli for the same sialylation-dependent molecular complexes [58].

Viral sialidases (neuraminidases) are crucial for the flu infection process. Structurally, influenza neuraminidases are divided into two phylogenetic subtypes, which are as follows: group 1 (N1, N4, N5, N8) and group 2 (N2, N3, N6, N7 and N9). Viral neuraminidase and hemagglutinin bind to the terminal sialic acid residues of the glycocalyx on the surface of the host cell for viral internalization by the host cell. Neuraminidase binds to sialic acid and cleaves α-(2,3) and α-(2,6) glycosidic bonds of terminal residues. Neuraminidase plays a key role in the infection and spread of the virus, so all the currently developed neuraminidase inhibitors are effective only when administered no later than 36–48 h after the first onset of symptoms [59,60]. The seasonal dynamics of sialidase activity (human and viral) in connection with the flu was studied, with the finding of an increased viral sialidase activity correlated with the severity of the disease. Elevated viral sialidase mRNA expression was observed alongside increased activity of human (endogenic) sialidase, hinting at the contribution of viral enzymes to overall atherogenicity. There is supportive evidence in the literature that viral infections aggravate cardiovascular conditions, such as myocardial infarction, heart failure, arrhythmia, myocarditis [61,62].

Sialidase is also present in erythrocyte membranes, with a marked increase in sialidase activity observed in stroke patients. The increased activity of sialidase leads to the cleavage of sialic groups located at the ends of complex carbohydrates of erythrocyte membranes, leading to an increase in the level of free sialic acid in blood plasma [63].

## 7. Trans-Sialidase Discovery

The discovery of atherogenic plasma activity characterized by decreased LDL sialylation led to establishing the enzyme carrying out the desialylating activity. The corresponding plasma enzyme was purified and its properties were studied. This 65 kDa enzyme with a specific affinity to LDL primarily catalyzes the hydrolysis of alpha-2,6 chemical bonds of terminal sialic acid residues, transferring them onto circulating plasma proteins [64]. Trans-sialidases remove and then transfer sialic acid residues between sialogalactosides. The highest rate of sialic acid residue transfer in vitro was observed for the α2-6 bond, and the lowest rate for the α2-8 bond [65].

The treatment of LDL with purified trans-sialidase results in the desialylation of LDL and accumulation of cholesterol esters in the human aortic intima smooth muscle cells. Thus, trans-sialidase may be involved in the formation of foam cells [64].

Sialidases (Neu) catalyze the removal of terminal sialic acid residues. Either class of enzymes can potentially participate in atherogenic LDL modification and serve as manifestations of blood atherogenicity. All human sialidases (NEU1–4) can exert their catalytic activities in circulation [66]. The desialylation of LDL in vitro by human Neu1 and Neu3 led to an increase in their atherogenicity in models of cultured human and mouse macrophages [67].

Neu inhibitor 2,3-dehydro-2-deoxy-2β-N-acetylneuraminic acid did not affect LDL desialylation in experiments, which supports the idea that desialylation is performed by trans-sialidase [24]. It should be noted that the amino acid sequence (and corresponding coding gene) of trans-sialidase [64] has not been determined yet, leaving intrigue (there is always a possibility that this activity belongs to the so-called ‘moonlight’ protein, which has never attracted attention in the field of sialidase studies) and space for future discoveries.

**Figure 2:**
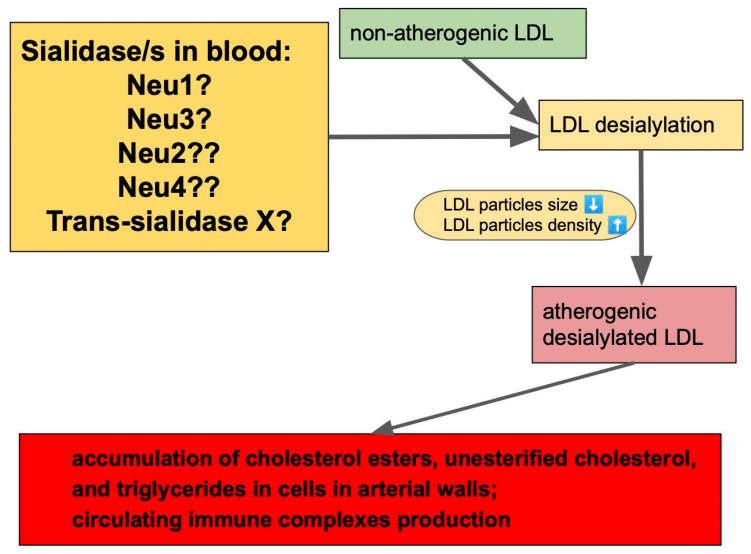
Potential contributors to sialidase activity in blood are neuraminidases (Neu1–4) and yet to be identified trans-sialidase. One of these enzymes (or a combination) leads to desialylation of normal LDLs, giving them atherogenic properties. “?” sign at neuraminidases means that it is not known exactly which one/s contributes to sialidase activity in blood. Arrows between boxes represent a sequence of events leading to atherogenic changes starting from enzymatic activities leading to the elimination of sialic acid residues from LDLs. Deasialilation of LDL also leads to a reduction in particle’s size, and increase in particle’s density.

All potential contributors to plasma sialidase activity are shown in Figure 2.

## 8. Animal Model Experiments

The reduction in Neu1 expression in *Neu1(hypo) Apoe^(^^−/−)^* mice led to the reduction in serum levels of VLDL, and LDL cholesterol, and caused the reduction in atherosclerotic lesions in comparison with *Apoe^(−/−)^* mice [51]. Moreover, treatment of *Apoe^(−/−)^* mice with pan-sialidase inhibitor (DANA) caused a significant reduction in atherosclerotic lesion development [51].

Recently, a further logical development of the approach used in the above-mentioned study was carried out. A novel pathway was described that takes place at early stages of atherosclerosis, involving desialylation of LDL in circulation [67]. It was hypothesized that sialidases (Neu) remove sialic acid residues from LDL glycoprotein and glycolipid components, thus, promoting atherosclerosis. Animal models of human atherosclerosis were obtained in *Neu4^(−/−)^*, *Neu3^(−/−)^*, and NEU1-deficient (*CathA^S190A-Neo^*) mice. Human enzymes Neu2, Neu3, Neu4 were expressed in *Escherichia coli* and then purified. Neu1 was expressed in HEK293 cells; transduced with a *CathA-IRES-NEU1* lentivirus. Sialidases were used for LDL modification. It was shown that ApoE-deficient mice injected with desialylated LDL demonstrated vascular accumulation of LDLs via lectin receptor Asgr1. In *Neu3^(−/−)^ Apoe(−/−)*, *Neu4^(−/−)^ Apoe^(−/−)^* and *CathA^S190A-Neo^ Apoe^(−/−)^* knockout mice, it was demonstrated that a 90% reduction in Neu1 activity or Neu3 complete inactivation diminished atherogenesis. In *Neu4^(−/−)^ Apoe^(−/−)^* mice, the atherogenesis rate was almost the same as in Neu4-intact *Apoe^(−/−)^* animals. *CathA^S190A-Neo^ Apoe^(−/−)^* mice showed decreased penetrability of their arteries compared to *Neu3^(−/−)^ Apoe^(−/−)^* and *Neu4^(−/−)^ Apoe^(−/−)^*, thus, pointing at potentially different mechanisms that regulate atherogenesis and inflammatory response. LDLs were shown to be desialylated by Neu1 and Neu3. Neu1 was shown to participate in the inflammatory response. The lipoprotein content analysis showed that plasma ApoB of *CathA^S190A-Neo^ Apoe^(−/−)^* mice was more sialylated, which is probably the factor influencing the atherogenesis rate in *CathA^S190A-Neo^ Apoe^(−/−)^* mice. The plasma LDL content in *Neu1^(−/−)^* mice was higher compared to the wild type (by 3-fold). Lowering Neu1 activity in *CathA^S190A-Neo^ Apoe^(−/−)^* mice slowed down atherosclerosis progression, while the LDL levels remained intact. This suggests that Neu1 governs LDL uptake [67]. The role of sialidase as a proatherogenic factor in vivo was studied in experiments with immobilized sialidase. It was found that a sialidase injection caused long-term reduction in LDL sialic acid content by 50% [57]. The selective inhibition of Neu1 and Neu3 in *LDLR^(−/−)^* and in *Apoe^(−/−)^* backgrounds of mice fed a high-fat diet achieved a significant reduction in atherosclerotic lesions, while the plasma levels of total cholesterol, triglycerides, LDL-C and HDL-C remained the same. Thus, the evidence points in the direction that in model conditions, Neu1 has pro-atherogenic and pro-inflammatory properties.

## 9. Putative Pharmacological Intervention, Future Directions of Research and Conclusions

The vast involvement of desialylation in atherosclerosis onset and progression opens a spectrum of opportunities for targeted pharmacological interventions. It has already been established that sialylation is a promising target for developing novel treatment strategies for influenza. A recombinant sialidase DAS181 derived from *Actinomyces viscosus*, if administered by inhalation, cleaves sialic acid from the respiratory epithelium, thereby inhibiting influenza virus binding [68]. C9-BA-DANA, sialidase inhibitor, reduced sialidase enzymatic activity in the lung epithelium, endothelium and fibroblasts, providing a potential way to manage sepsis and atherosclerosis [69]. One could assume that once the exact sequence and corresponding gene of plasma trans-sialidase is figured out, a potential new antiatherosclerotic therapy could be developed by the creation of specific trans-sialidase inhibitors, leading to the reduction in LDLs with reduced sialylation in blood plasma, and thus, to the reduction in atherosclerosis progression. In addition, one of the future directions of research should probably be shifted towards an attempt to connect together the different aspects of atherosclerosis pathology. For example, to find out if there is any possible relation between the mutations of mitochondrial DNA shown earlier to be associated with atherosclerosis [70] and modified LDL (including the desialylated ones). The tremendous work by V. Tertov allowed us to move closer to an understanding of the role of sialylation in atherosclerosis development, and to the creation of new ways to prevent this pathology.
